# A new m^6^A methylation-related gene signature for prognostic value in patient with urothelial carcinoma of the bladder

**DOI:** 10.1042/BSR20204456

**Published:** 2021-04-09

**Authors:** Bin Zheng, Jianwei Wang, Guiting Zhao, Xiaoxu Chen, Zhongshun Yao, Zhihong Niu, Wei He

**Affiliations:** 1 Cheeloo College of Medicine, Shandong University, Jinan, Shandong, P.R. China; 2 Department of Urology, Shandong Provincial Hospital Affiliated to Shandong First Medical University, Jinan, Shandong, P.R. China; 3 Department of Urology, Shandong Provincial Hospital Affiliated to Shandong University, Jinan, Shandong, P.R. China; 4 Department of Urology, Shandong Provincial ENT Hospital Affiliated to Shandong University, Jinan, Shandong, P.R. China; 5 Department of Pediatric Surgery, The First People’s Hospital of Jining city, Jining, Shandong, P.R. China

**Keywords:** m6A methylation, m6A-related genes, prognostic signature, survival, urothelial carcinoma of the bladder

## Abstract

Background: Bladder cancer (BC) is one of the most common malignant urological cancer in the world. Because of its characteristic of easy-recurrence and muscle-invasive, advances in our genetic understanding of bladder cancer should be translated into prognostic indicators.

Methods: We investigated 16 m^6^A RNA methylation regulators from The Cancer Genome Atlas (TCGA) database and The Human Protein Atlas (HPA) database. The expression profile, clinical application as well as prognostic value of these genes in UC were investigated. Moreover, we further explored the correlation between RNA methylation genes and biological functions, pathways and immune status.

Results: Five m^6^A-related genes (*HNRNPC, YTHDF2, YTHDF1, HNRNPA2B1, METTL3*) up-regulated in UC tissues, while three regulators (*ZC3H13, METTL16, FTO*) down-regulated in UC. *FTO* and *YTHDF2* show biomarker potential for the prognosis of UC patients. In addition, these identified genes may related with essential functions and core molecular pathways.

Conclusions: Our research shows that two m^6^A RNA methylation regulators can serve as reliable prognostic biomarkers of UC, which might be exerted as potential targets of therapeutic strategies.

## Introduction

Currently, bladder cancer (BC) is the fourth most common diagnosed cancer in men and the most common type of cancer among urinary cancers, with approximately 61,700 new cases and 12,870 deaths in the United States [1]. Among various pathological types of bladder cancer, urothelial carcinoma (UC) is the predominant phenotype of bladder cancer, with a minority of tumors such as squamous, plasmacytoid, sarcomatoid and so on [2]. Until recently, the treatment for BC remain limited, including surgery, chemo-radiotherapy, immunotherapy, etc. The limited number of treatments contributed to little progress, with a flat 5-year survival rate [3,4]. Significantly, up to 25% of new diagnosed cases present as muscle-invasive or advanced stage (T2-T4),with a very poor prognosis [5]. Therefore, it is meaningful for us to find out high accurate prognostic biomarkers, which could help patients with optimal individual treatments.

Recently, 182 different RNA modifications have been found according to MODOMICS [6]. The chemical modifications into RNA plays a vital role in regulating the post-transcription of gene expressions programs during development. Accordingly, altered RNA modification are widely related to diseases [7]. Many studies showed that the dysregulation of N^6^-methyladenosine (m^6^A) modification emerged as a contributor to different cancers, such as pancreatic cancer, renal cell cancer and BC [8–10]. m^6^A is the most common chemical modification of RNA in eukaryotes [11]. The deposit of m^6^A requires three types of proteins, first, methyltransferases called writers (METTL3, METTL14, WTAP, etc.), second, binding proteins called readers (YTHDF1, YTHDF2, YTHDC1, etc.), third, demethylases called erasers (FTO and ALKBH5). It has been reported that m^6^A regulators may act as different or even contradictory role in diverse cancers [12–14]. However, the precise correlation between m^6^A genes and its value of prognosis in UC are not clear as yet.

Here, we reported the expression pattern of 16 m^6^A-related genes in 411 tumor and 19 normal samples of UC in The Cancer Genome Atlas (TCGA) datasets. Then, we divided UC patients with different prognosis into two clusters by consensus clustering analysis. The correlation among the m^6^A-related gene and its relationship with clinical characteristics were analyzed as well. Besides, we conducted univariate, LASSO and multivariate Cox regression analysis. Based on these results, risk stratification models were constructed. Finally, the prognostic value of the gene risk signature was validated in TCGA test cohort as well as the HPA database.

## Materials and methods

### Dataset acquisition and analysis

The RNA-seq data and clinicopathological information of bladder urothelial carcinoma were downloaded from the TCGA database (https://portal.gdc.cancer.gov/). The immunohistochemical (IHC) images were downloaded from The HPA database. All gene expression data have been normalized by “limma” package.

After performing a comprehensive literature review, we identified 16 m^6^A RNA methylation genes from previous literature (*METTL3, METTL14, METTL16, YTHDF1, YTHDF2, YTHDF3, WTAP, KIAA1429, YTHDC1, YTHDC2, RBM15, ZC3H13, FTO, ALKBH5, HNRNPA2B1 HNRNPC*) [13–16].

We used “limma”, “pheatmap” and “vioplot” package to assess and visualize the expression level of m^6^A-related genes separately. The co-expression correlation analysis was visualized by “corrplot” package. The protein–protein interaction (PPI) network of all m^6^A-related genes was performed by the STRING database (http://string-db.org/).

The “ConsensusClusterPlus” was utilized to categorized patients. The principal component analysis (PCA) was performed to confirm diverse m^6^A-related genes expression patterns in diverse UC groups, and the clinicopathologic features between UC groups was visualized by “pheatmap” package.

### Construction and validation of the prognostic signatures

To screen the prognostic-related genes as many as possible, we performed the univariate Cox analysis with *P*<0.15. After filtering out prognosis-related differentially expressed genes (DEGs) with “venn” package, we performed LASSO regression analysis and Multivariate Cox regression analyses to identify the optional prognostic genes for the model. All UC patients (in train- and test set) were stratify into high- and low-risk sets by median risk score. The Kaplan–Meier curve and receiver operating characteristic (ROC) curve were used to evaluate the accuracy of the prognostic model. The Human Protein Atlas database (HPA) (https://www.proteinatlas.org/) was utilized to validate this model as well. The univariate and multivariate Cox analysis of clinical characteristics was performed to analyze independent prognostic factors.

### Functional analyses

**Figure 1:**
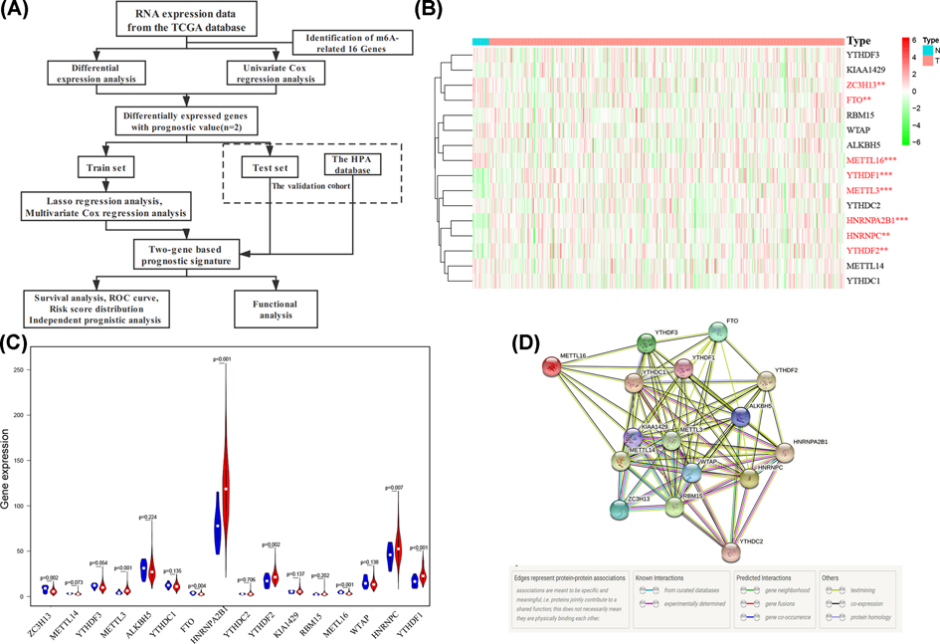
The flow chart and expression pattern of 16 m^6^A RNA methylation genes (**A**) The flow chart of this study. (**B** and **C**) The heatmap and violin plot of 16 m^6^A RNA methylation genes compared with UC samples and normal samples. (**D**) The PPI network of 16 m^6^A-related genes; *P<0.05, **P<0.01, ***P<0.001.

Gene Ontology (GO) and Kyoto Encyclopedia of Genes and Genomes (KEGG) analyses based on the DEGs (|log_2_FC| ≥ 1, FDR < 0.05) were conducted by “clusterProfiler” package. Single-sample gene set enrichment analysis (ssGSEA) was performed by the “gsva” package [17]. The flow chart is shown in Figure 1A.

### Statistical analysis

Mann–Whitney test was utilized to measure gene expression between UC tissues and non-tumor tissues. Samples with incomplete clinical information were eliminated. The K–M curve with a log-rank test was adopted. All data in the present study were analyzed with the R statistical package (R version 4.0.1). A two-tailed *P*<0.05 was considered significant.

## Results

### The expression pattern and correlation of m^6^A methylation genes

Heatmap and violin plot were performed to analyze the expression pattern of m^6^A methylation genes between UC and normal samples (Figure 1B,C). Genes up-regulated in UC tissues are *HNRNPC* (*P*=0.007), *YTHDF2* (*P*=0.002), *YTHDF1* (*P*<0.001), *HNRNPA2B1* (*P*<0.001) and *METTL3* (*P*<0.001). While 3 out of 16 genes show relatively low expression level, including *ZC3H13* (*P*=0.002), *METTL16* (*P*<0.001) and *FTO* (*P*=0.004). Significant difference was not observed in *WTAP* (*P*=0.138), *YTHDF3* (*P*=0.054), *METTL14* (*P*=0.073), *YTHDC1* (*P*=0.135) and *ALKBH5* (*P*=0.224). The correlation analysis reveals that *YTHDF3* and *KIAA1429* are most relevant (*r* = 0.67). Moreover, *KIAA1429* and *METTL14* are respectively correlated with the other 15 genes (Supplementary Figure S1A). The PPI network of the 16 genes shows that *YTHDAC1, KIAA1429, METTL3, WTAP, ALKBH5* and *HNRNPC* are hub genes (Figure 1D).

### Consensus clustering analysis of m^6^A methylation genes identified two clusters in UC

**Figure 2:**
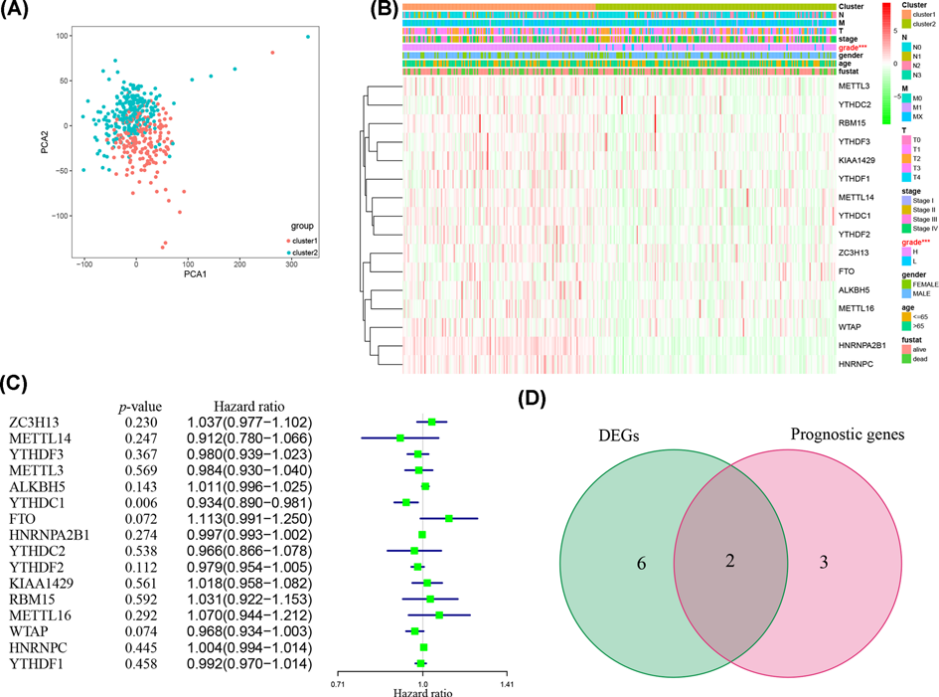
Expression pattern and clinical outcome in two clusters (**A**) Principal component analysis plot of UC patients in the TCGA database. (**B**) The heatmap of clinicopathologic features and the expression of m^6^A methylation genes between two clusters. (**C**) Univariate Cox regression regarding OS. (**D**) Venn plot indicates the prognosis-related DEGs.

Due to relatively small number of cases in one of clusters and the need to ensure the maximum intra-group correlation and minimum inter-group correlation, we divided all samples into two sets (cluster 1 and 2) (Supplementary Figures S2A and 2B). Then, the result of PCA further confirms the accuracy about clustering results (Figure 2A). Afterwards, the clinicopathological characteristics indicate significant difference for the tumor grade (*P*<0.001) (Figure 2B). However, other characteristics, such as tumor stage, gender, age, etc., did not vary significantly between two clusters. These results suggest that m^6^A methylation genes are related to tumor malignancy of UC.

### Construction of m^6^A-related genes-based prognostic signature

**Figure 3:**
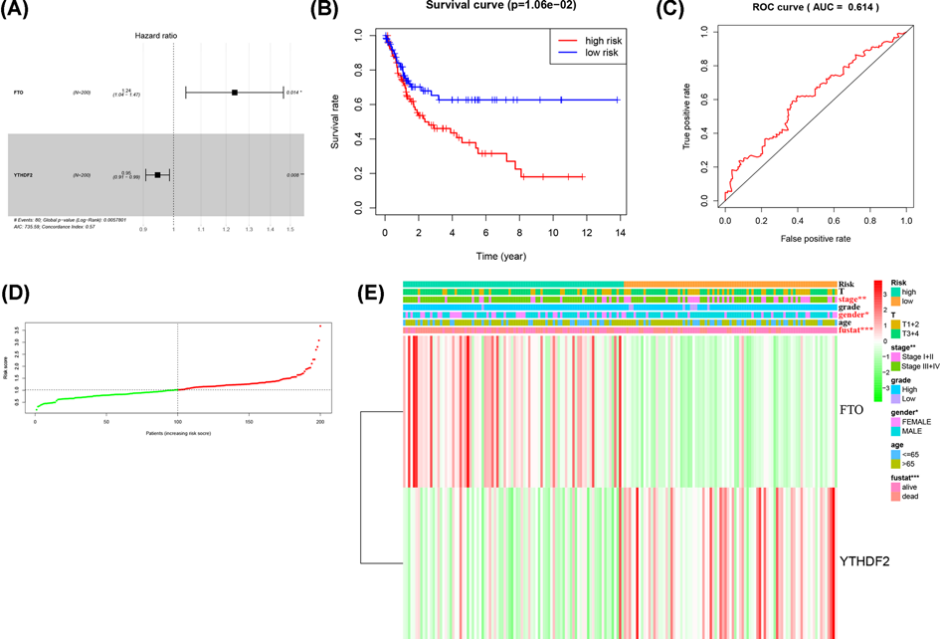
Construction of prognostic risk signature with two m^6^A-related genes (**A**) Multivariate Cox analysis of the selected genes. (**B**) The survival analysis of two subgroups. (**C**) The ROC curve to evaluate the prediction efficiency of the prognostic signature. (**D**) Heatmap shows clinicopathologic features of two groups. (**E**) The distributions of risk scores in train set. The red and green dots represent high-risk patients and low-risk patients, separately.

To investigate the prognostic value of m^6^A methylation genes, the entire TCGA cohort with necessary clinical information was divided into train- (*n*=200) and test group (*n*=200). Then, we conducted the univariate Cox regression in TCGA train set to further validate the prognostic value of m^6^A-related genes. The results reveal that the expression of *FTO* and *ALKBH5* are correlate with worse survival rates and the expression of *YTHDC1, WTAP* and *YTHDF2* are correlate with better survival rates (Figure 2C). After identifying two DEGs that are correlated with OS with Venn diagram (Figure 2D), we performed LASSO regression analysis, multivariate Cox regression analysis and finally built the prognostic signature by *FTO* (HR = 1.24, 95%CI [1.04–1.47], *P*=0.014) and *YTHDF2* (HR = 0.95, 95%CI [0.91–0.99], *P*=0.008) (Figure 3A; Supplementary Figure S2C and S2D). Afterwards, coefficients were applied to calculate each UC patients’ risk scores with the following formula: risk score = (-0.056) × YTHDF2 + (0.207) × FTO.

After dividing all UC samples into high- and low risk set by median risk score, we performed the K–M analysis, which presents that high-risk patients have worse OS than patients in the low risk set in train set (*P*<0.05) (Figure 3B). Then, the ROC curve demonstrates that the prognostic signature has an acceptable efficiency (AUC = 0.614) (Figure 3C). What’s more, the distribution of risk scores confirms that patients with high-risk scores have poor survival outcomes as well (Figure 3D). A heatmap was made to illustrate the difference of two m^6^A-related genes between two groups, and the results show that tumor stage (*P*<0.01), gender (*P*<0.05) and survival state (*P*<0.001) have significant statistic difference. As shown in Figure 3E and Supplementary Figure S2A, overall, the results demonstrate that the two-gene risk signature could effectively filter out high risk UC patients with poor clinic outcomes.

### Validation of the prognostic signature

**Figure 4:**
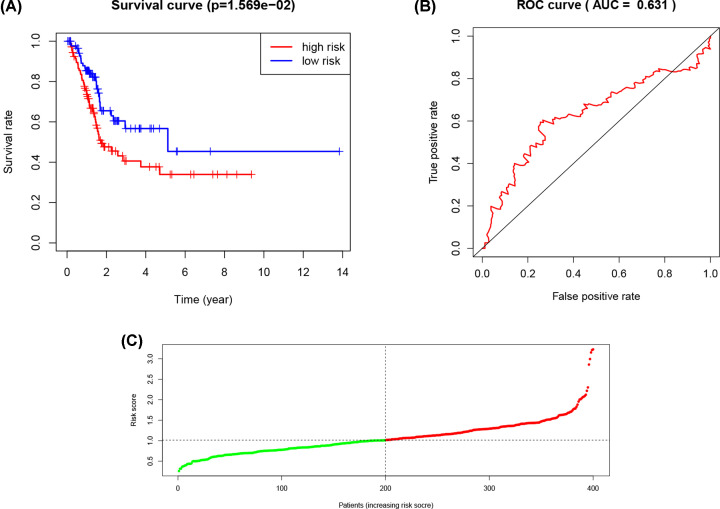
Validation of the risk model in the test set (**A**) The K–M curves for OS of UC patients. (**B**) The ROC curve in test set. (**C**) The distribution of risk scores. The red and green dots represent high-risk patients and low-risk patients, separately.

Based on train set’s median risk scores, patients in test set were classified into high- and low risk set. We performed K–M curve, ROC curve and the risk scores distributions as well. The results show that patients in high risk group had worse OS compared with patients in low risk group in test group (*P*<0.05) (Figure 4A). Besides, the ROC curve indicates that risk score has a relatively high predictive ability (AUC = 0.631) (Figure 4B). The distribution of risk scores also demonstrates that patients with high risk scores have higher mortality in test group (Figure 4C and Supplementary Figure S1C).

**Figure 5:**
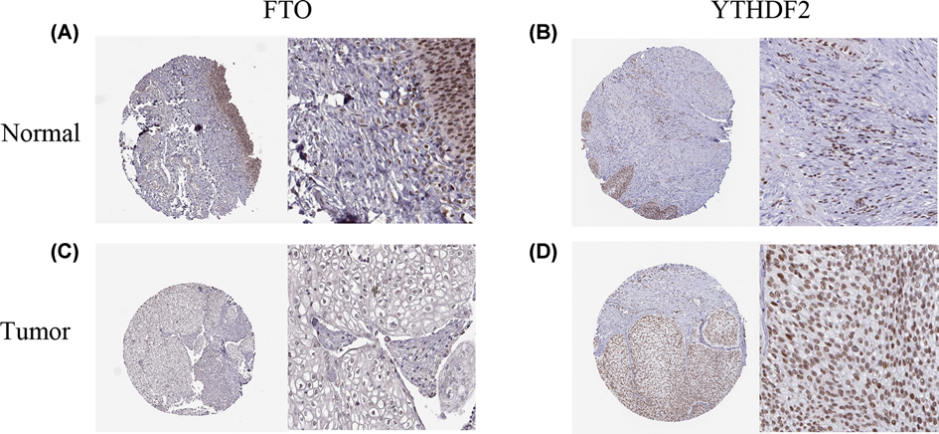
Validation of the risk model in the HPA database The IHC expression pattern of FTO (**A** and **C**) and YTHDF2 (**B** and **D**) in normal tissue and tumor tissue.

In order to further validate the two-gene model and to simulate clinic applications, we made use of the HPA database to assess IHC pattern. As shown in Figure 5A,C, we noticed that normal bladder tissue staining of *FTO* exhibited higher staining than UC samples. On the contrary, for YTHDF2, the strong staining was observed in UC samples (Figure 5B,D). These IHC staining forms may assist in distinguishing tumor from normal tissues

### Independent prognostic value of the two-gene signature in UC

**Figure 6:**
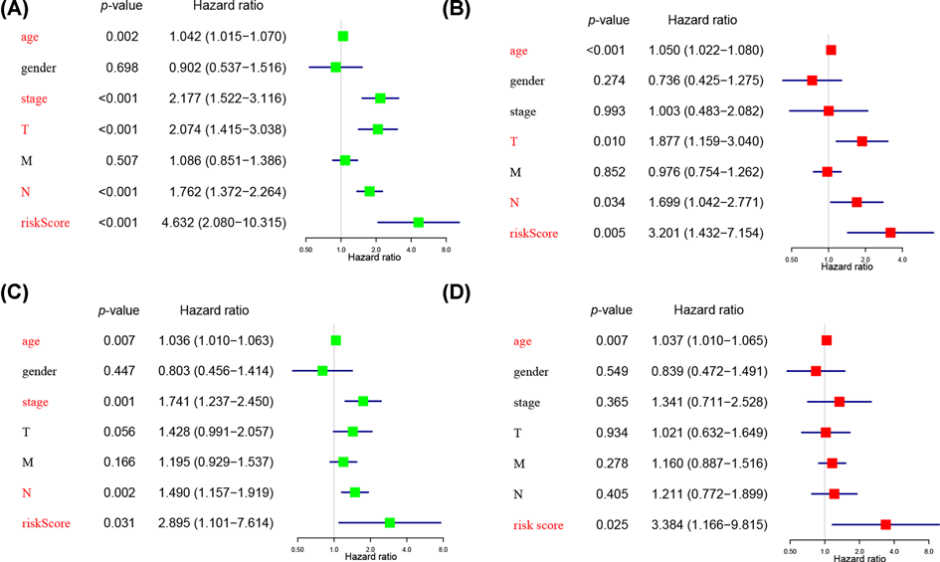
Results of the independent prognostic factors in train and test set (**A** and **C**) Univariate Cox analyses regarding OS in train (A) and test set (C). (**B** and **D**) Multivariate Cox analyses regarding OS in train (B) and test set (D).

To confirm whether risk score could serve as independent prognostic biomarker, we carried out the univariate and multivariate Cox regression analysis both in train- and test set (Figure 6A–D). Both the multivariate and univariate Cox regression analysis show that risk score is significantly associated with poor OS in train set (HR = 3.201, 95% CI [1.432–7.154], *P*=0.005) as well as test set (HR = 3.384, 95% CI [1.166–9.815], *P*=0.025) (Figure 6A–D). Besides, in train set, clinic characteristics, such as age (HR = 1.050, 95% CI [1.022–1.080], *P*<0.001), tumor T stage (HR = 1.877, 95% CI [1.159–3.040], *P*=0.010), N stage (HR = 1.699, 95% CI [1.042–2.771], *P*=0.034) also serve as independent prognostic factors (Figure 6B). Taken together, these results verify that the two m^6^A-related genes prognostic model has accurate prognostic value.

### Functional analysis

**Figure 7:**
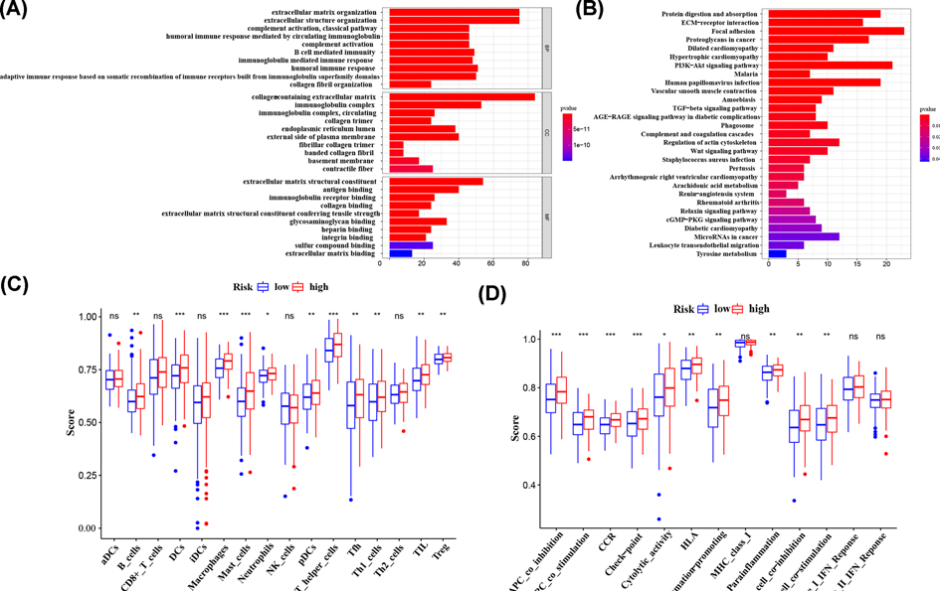
Representative results of functional analyses (**A** and **B**) Representative results of the most significant GO enrichment and KEGG pathways. (**C**) The scores of 16 immune cells. (**D**) The scores of 13 immune-related functions; ns, not significant; *P<0.05, **P<0.01, ***P<0.001.

We also elucidated the functional analysis based on the differentially expression genes. By performing GO enrichment, we found that these genes mainly enriched in two types of biological functions: extracellular matrix (ECM)-related functions, such as extracellular matrix organization, extracellular structure organization and immune-related functions, including B cell–mediated immunity, humoral immune response and so on (*P*<0.05; Figure 7A). For KEGG, the associated networks are enriched in numerous of classic pathways, for instance, PI3K-Akt signaling pathway, Wnt signaling pathway and TGF-β signaling pathway (*P*<0.05; Figure 7B). Considering its potential role in immune-related functions, we further investigated the correlation between risk score and immune status with ssGSEA. Interestingly, the score of the antigen presentation process and T-cell related functions, for example, DCs, pDCs, APC co-stimulation, HLA I and T-helper cells, Th1 cells, T-cell co-inhibition, were higher in high risk set (*P*<0.05; Figure 7C,D).

## Discussion

The m^6^A methylation is the most abundant mRNA modification and has exerted as a widespread regulatory functions in various pathological processes [18]. Many studies has found that several m^6^A sites could be regulated in a disease-specific manner and the modification of m^6^A methylation existed in responding to various signaling pathways [19]. Bladder cancer, as a prevalent disease, has a high rate of morbidity and mortality as well as the huge cost. The development of BC is generated via two different types, developing from papillary NMIBCs or non-papillary MIBCs [20]. Most of patients are diagnosed by painless hematuria or microscopic hematuria. After adequate evaluations with cystoscopy or imaging examination, different treatments, including transurethral resection of bladder tumor (TURBT), radical cystectomy, adjuvant intravesical therapy or adjuvant therapy will be performed [21]. However, due to lacks of accurate molecular biomarkers for timely diagnosis and prognosis assessment, bladder cancer remains a lethal disease and numbers of patients still experience recurrence or led to unsatisfactory results [21].

In the present study, we found that 8 out of 16 genes were abnormally expressed in UC. Among these genes, many of them had a negative relation with the malignancy of UC, which implied its distinct functions in UC. Based on the expression pattern of these m^6^A-related genes, the TCGA UC cohort was divided into two clusters with significantly statistic difference for tumor grade. What’s more, we also conducted a two-gene prognostic signature, which shows a good ability for predicting the clinical outcomes of UC. Besides, risk score, age, T stage and N stage successfully serve as independent prognostic factors with relatively high HR value. Finally, we successfully validated this risk signature with TCGA test set. Because our results present that the prognostic signature is positive with the OS survival analysis, which implies that these genes on the molecular level have potential clinical value, we further validate the value of clinical application through the HPA database. The IHC staining demonstrates that *FTO* and *YTHDF2* may be potentially ideal prognosis biomarker and therapeutic target for UC patients.

We also performed GO and KEGG pathway enrichment analyses and illustrated the interaction between risk score and immune status in TCGA cohort. For KEGG pathways, these genes enrich in PI3K-Akt signaling pathway, Wnt signaling pathway and TGF-β signaling pathway, which are key drivers of UC [22]. It has been reported that hyper-activated PI3K-Akt signaling pathway acts as essential regulator of aerobic glycolysis in BC, which makes cancer metabolic switch and cell proliferation [23]. Moreover, in UC, both Wnt signaling pathway and TGF-β signaling pathway play crucial role in the process of EMT, hence regulating cell invasion and metastasis [24–26]. Besides, our findings for the two-gene prognostic signature regarding its functions indicates that these genes link to ECM-related functions and immune-related functions. Previous data suggest that dysregulation of tumor ECM has a principal role in onset as well as progression of BC, both at primary and metastatic sites [27]. Remodeled ECM or changed form of ECM structures can even induce cell proliferation, migration and inflammation [28,29].

Apart from several classic functions and pathways, we found that several tumor immune-related cells and functions were enriched as well. By using the ssGSEA, the score of macrophages and DCs in high-risk group were higher than low-risk UC group (*P*<0.001) and cellular immune-related cells, such as B-cell and T cell-related immune cells also had higher score than low-risk group (*P*<0.01). Some published data show that some m^6^A -associated proteins can regulate antitumor immune responses. Recent studies uncovered that *FTO* and *YTHDF2* could affect M1 and M2 macrophage activation by regulating its polarization [30–32]. Specifically, through *YTHDF2* involvement, low expressed *FTO* inhibits NF-κB pathway and further decreases the stability of PPAR-γ, which impedes macrophage activation [32]. In addition, Su et al. found that FTO could elevate the expression of *LILRB4*, an immune checkpoint regulator that can inhibit T cell activity and stimulate tumor infiltration [33]. Published data have reported that immune check-points was an important mechanism of tumor immune evasion [34]. Therefore, *FTO* may play a potential role in managing immune evasion and sensitizing tumor cell to T cell cytotoxicity by m^6^A-dependent mechanism. Moreover, Yang et al. suggest that through m^6^A “reader” YTHDF2-mediated mRNA decay, suppressed *FTO* inhibits tumorgenicity as well as the expression of PD-1, and sensitizes tumor cells to anti-PD-1 blockade, which provides novel sight to reduce drug resistance [35]. Meanwhile, m^6^A -related proteins impact B-cell and T-cell development as well. Some studies found that the loss of METTL14 in B-cell could lead severe defects in B-cell development, specifically in the process of large pre-B-cell to small pre-B-cell [36,37]. In another study, Li et al. found that the absence of METTL3 in native T-cell could decrease the number of Th1 and Th17 cells and increase Th2 cells [38]. Apparently, our results consistent with previous studies.

Previous study has been discovered that reader YTHDF2 can alter cell migration in BC. Xie et al. found that the depletion of aberrantly expressed *YTHDF2* could suppress the capability of mRNA degradation (including tumor suppressor *SETD7* and *KLF4*) and impede cell migration *in vivo* and *in vitro* via recognition of METTL3-catalyzed sites, which suggest that the important role of *YTHDF2* in progression of BC may work through *METTL3*/ *YTHDF2/SETD7/KLF4* m^6^A axis [39]. Eraser FTO is also a critical player in BC. Wen et al found that knockdown of *FTO* apparently induces BC cell proliferation and migration, and the effect of cisplatin-induced cytotoxicity of BC cell is rescued via the inhibition of *FTO* [40]. In addition, FTO can demethylate *MALAT1* which further promote BC cell viability and proliferation through the *miR-384/MAL2* m^6^A axis [41]. Similarly, Song et al. revealed that FTO, stabilized by USP-18 via inhibition of proteasomal degradation, reduces *PYCR1* m^6^A methylation and stabilizes its transcript, which finally facilitates BC cell initiation and progression [42]. Taken together, these results confirm the efficiency of this prognostic model.

However, we admitted that there are also several disadvantages during our analyses. First, the UC cohort is relatively small and clinical data of samples is also not complete. Second, lacks of *in vitro* and *in vivo* experimental verification also affects the rigor of this article. Therefore, we will collect the data of UC patients treated in our center and verify our finding *in vivo* and *in vitro* experiments.

## Conclusions

Our study indicates a dysregulated expression pattern of m^6^A methylation genes between UC samples and normal samples. Besides, the risk model based on selected m^6^A-related genes accurately distinguishes UC patients with diverse prognosis and the Cox hazard model shows that risk score, age, T and N stage are significantly associated with patients’ prognosis, which suggests a pivotal role of m^6^A-related genes in the tumorigenesis and progression of UC.

## Supplementary Material

Supplementary Figure S1-S2

## Data Availability

All data are available from the sources listed in the manuscript—the TCGA data portal and the HPA database.

## Abbreviations

BC: bladder cancer
DEG: differentially expressed gene
ECM: extracellular matrix
HPA: The Human Protein Atlas
PPI: protein–protein interaction
TCGA: The Cancer Genome Atlas

## References

[1] Siegel R.L., Miller K.D. and Jemal A. (2019) Cancer statistics, 2019. CA Cancer J. Clin. 69, 7–34 10.3322/caac.21551 30620402

[2] Wang L., Smith B.A., Balanis N.G., Tsai B.L., Nguyen K., Cheng M.W. et al. (2020) A genetically defined disease model reveals that urothelial cells can initiate divergent bladder cancer phenotypes. Proc. Natl. Acad. S 117, 563–572 10.1073/pnas.1915770117 31871155 PMC6955337

[3] Pierantoni F., Maruzzo M., Gardi M., Bezzon E., Gardiman M.P., Porreca A. et al. (2019) Immunotherapy and urothelial carcinoma: an overview and future prospectives. Crit. Rev. Oncol. Hematol. 143, 46–55 10.1016/j.critrevonc.2019.08.005 31476551

[4] Grayson M. (2017) Bladder cancer. Nature 551, S33 10.1038/551S33a 29117156

[5] Chang S.S., Bochner B.H., Chou R., Dreicer R., Kamat A.M., Lerner S.P. et al. (2017) Treatment of non-metastatic muscle-invasive bladder cancer: AUA/ASCO/ASTRO/SUO Guideline. J. Urol. 198, 552–559 10.1016/j.juro.2017.04.086 28456635 PMC5626446

[6] Boccaletto P., Machnicka M.A., Purta E., Piatkowski P., Baginski B., Wirecki T.K. et al. (2018) MODOMICS: a database of RNA modification pathways. 2017 update. Nucleic Acids Res. 46, D303–D307 10.1093/nar/gkx1030 29106616 PMC5753262

[7] Delaunay S. and Frye M. (2019) RNA modifications regulating cell fate in cancer. Nat. Cell Biol. 21, 552–559 10.1038/s41556-019-0319-0 31048770

[8] Green N.H., Galvan D.L., Badal S.S., Chang B.H., LeBleu V.S., Long J. et al. (2019) MTHFD2 links RNA methylation to metabolic reprogramming in renal cell carcinoma. Oncogene 38, 6211–6225 10.1038/s41388-019-0869-4 31289360 PMC8040069

[9] Zhang J., Bai R., Li M., Ye H., Wu C., Wang C. et al. (2019) Excessive miR-25-3p maturation via N(6)-methyladenosine stimulated by cigarette smoke promotes pancreatic cancer progression. Nat. Commun. 10, 1858 10.1038/s41467-019-09712-x 31015415 PMC6478927

[10] Jin H., Ying X., Que B., Wang X., Chao Y., Zhang H. et al. (2019) N(6)-methyladenosine modification of ITGA6 mRNA promotes the development and progression of bladder cancer. EBioMed. 47, 195–207 10.1016/j.ebiom.2019.07.068 31409574 PMC6796523

[11] Meyer K.D., Saletore Y., Zumbo P., Elemento O., Mason C.E. and Jaffrey S.R. (2012) Comprehensive analysis of mRNA methylation reveals enrichment in 3′ UTRs and near stop codons. Cell 149, 1635–1646 10.1016/j.cell.2012.05.003 22608085 PMC3383396

[12] Liu J., Yue Y., Han D., Wang X., Fu Y., Zhang L. et al. (2014) A METTL3-METTL14 complex mediates mammalian nuclear RNA N6-adenosine methylation. Nat. Chem. Biol. 10, 93–95 10.1038/nchembio.1432 24316715 PMC3911877

[13] Lan Q., Liu P.Y., Haase J., Bell J.L., Hüttelmaier S. and Liu T. (2019) The critical role of RNA m(6)A methylation in cancer. Cancer Res. 79, 1285–1292 10.1158/0008-5472.CAN-18-2965 30894375

[14] Chen X.Y., Zhang J. and Zhu J.S. (2019) The role of m(6)A RNA methylation in human cancer. Mol. Cancer 18, 103 10.1186/s12943-019-1033-z 31142332 PMC6540575

[15] Pan Y., Ma P., Liu Y., Li W. and Shu Y. (2018) Multiple functions of m(6)A RNA methylation in cancer. J. Hematol. Oncol. 11, 48 10.1186/s13045-018-0590-8 29587823 PMC5870302

[16] Zhao W., Qi X., Liu L., Ma S., Liu J. and Wu J. (2020) Epigenetic regulation of m(6)A modifications in human cancer. Mol. Ther. Nucleic Acids 19, 405–412 10.1016/j.omtn.2019.11.022 31887551 PMC6938965

[17] Rooney M.S., Shukla S.A., Wu C.J., Getz G. and Hacohen N. (2015) Molecular and genetic properties of tumors associated with local immune cytolytic activity. Cell 160, 48–61 10.1016/j.cell.2014.12.033 25594174 PMC4856474

[18] Zaccara S., Ries R.J. and Jaffrey S.R. (2019) Reading, writing and erasing mRNA methylation. Nat. Rev. Mol. Cell Biol. 20, 608–624 10.1038/s41580-019-0168-5 31520073

[19] McIntyre A.B.R., Gokhale N.S., Cerchietti L., Jaffrey S.R., Horner S.M. and Mason C.E. (2020) Limits in the detection of m(6)A changes using MeRIP/m(6)A-seq. Sci. Rep. 10, 6590 10.1038/s41598-020-63355-3 32313079 PMC7170965

[20] Sanli O., Dobruch J., Knowles M.A., Burger M., Alemozaffar M., Nielsen M.E. et al. (2017) Bladder cancer. Nat. Rev. Dis. Primers 3, 17022 10.1038/nrdp.2017.22 28406148

[21] Chang S.S., Boorjian S.A., Chou R., Clark P.E., Daneshmand S., Konety B.R. et al. (2016) Diagnosis and treatment of non-muscle invasive bladder cancer: AUA/SUO Guideline. J. Urol. 196, 1021–1029 10.1016/j.juro.2016.06.049 27317986

[22] Knowles M.A. and Hurst C.D. (2015) Molecular biology of bladder cancer: new insights into pathogenesis and clinical diversity. Nat. Rev. Cancer 15, 25–41 10.1038/nrc3817 25533674

[23] Massari F., Ciccarese C., Santoni M., Iacovelli R., Mazzucchelli R., Piva F. et al. (2016) Metabolic phenotype of bladder cancer. Cancer Treat. Rev. 45, 46–57 10.1016/j.ctrv.2016.03.005 26975021

[24] Zhou Q., Chen S., Lu M., Luo Y., Wang G., Xiao Y. et al. (2019) EFEMP2 suppresses epithelial-mesenchymal transition via Wnt/β-catenin signaling pathway in human bladder cancer. Int. J. Biol. Sci. 15, 2139–2155 10.7150/ijbs.35541 31592144 PMC6775297

[25] Wu G., Weng W., Xia P., Yan S., Zhong C., Xie L. et al. (2021) Wnt signalling pathway in bladder cancer. Cell. Signal. 79, 109886 10.1016/j.cellsig.2020.109886 33340660

[26] Chen Z., He S., Zhan Y., He A., Fang D., Gong Y. et al. (2019) TGF-β-induced transgelin promotes bladder cancer metastasis by regulating epithelial-mesenchymal transition and invadopodia formation. EBioMed. 47, 208–220 10.1016/j.ebiom.2019.08.012 31420300 PMC6796540

[27] Alfano M., Canducci F., Nebuloni M., Clementi M., Montorsi F. and Salonia A. (2016) The interplay of extracellular matrix and microbiome in urothelial bladder cancer. Nat. Rev. Urol. 13, 77–90 10.1038/nrurol.2015.292 26666363 PMC7097604

[28] Toole B.P. (2004) Hyaluronan: from extracellular glue to pericellular cue. Nat. Rev. Cancer 4, 528–539 10.1038/nrc1391 15229478

[29] de la Motte C.A., Hascall V.C., Drazba J., Bandyopadhyay S.K. and Strong S.A. (2003) Mononuclear leukocytes bind to specific hyaluronan structures on colon mucosal smooth muscle cells treated with polyinosinic acid:polycytidylic acid: inter-alpha-trypsin inhibitor is crucial to structure and function. Am. J. Pathol. 163, 121–133 10.1016/S0002-9440(10)63636-X 12819017 PMC1868154

[30] Liu Y., Liu Z., Tang H., Shen Y., Gong Z., Xie N. et al. (2019) The N(6)-methyladenosine (m(6)A)-forming enzyme METTL3 facilitates M1 macrophage polarization through the methylation of STAT1 mRNA. Am. J. Physiol. Cell Physiol. 317, C762–C775 10.1152/ajpcell.00212.2019 31365297

[31] Raggi F., Pelassa S., Pierobon D., Penco F., Gattorno M., Novelli F. et al. (2017) Regulation of human macrophage M1-M2 polarization balance by hypoxia and the triggering receptor expressed on myeloid cells-1. Front. Immunol. 8, 1097 10.3389/fimmu.2017.01097 28936211 PMC5594076

[32] Gu X., Zhang Y., Li D., Cai H., Cai L. and Xu Q. (2020) N6-methyladenosine demethylase FTO promotes M1 and M2 macrophage activation. Cell. Signal. 69, 109553 10.1016/j.cellsig.2020.109553 32018056

[33] Su R., Dong L., Li Y., Gao M., Han L., Wunderlich M. et al. (2020) Targeting FTO suppresses cancer stem cell maintenance and immune evasion. Cancer Cell. 38, 79.e11–96.e11 10.1016/j.ccell.2020.04.017 32531268 PMC7363590

[34] Beatty G.L. and Gladney W.L. (2015) Immune escape mechanisms as a guide for cancer immunotherapy. Clin. Cancer Res. 21, 687–692 10.1158/1078-0432.CCR-14-1860 25501578 PMC4334715

[35] Yang S., Wei J., Cui Y.H., Park G., Shah P., Deng Y. et al. (2019) m(6)A mRNA demethylase FTO regulates melanoma tumorigenicity and response to anti-PD-1 blockade. Nat. Commun. 10, 2782 10.1038/s41467-019-10669-0 31239444 PMC6592937

[36] Zheng Z., Zhang L., Cui X.L., Yu X., Hsu P.J., Lyu R. et al. (2020) Control of early B cell development by the RNA N(6)-methyladenosine methylation. Cell Reports 31, 107819 10.1016/j.celrep.2020.107819 32610122 PMC7371152

[37] Zhang W., He X., Hu J., Yang P., Liu C., Wang J. et al. (2019) Dysregulation of N(6)-methyladenosine regulators predicts poor patient survival in mantle cell lymphoma. Oncol. Lett. 18, 3682–3690 10.3892/ol.2019.10708 31516580 PMC6732954

[38] Li H.B., Tong J., Zhu S., Batista P.J., Duffy E.E., Zhao J. et al. (2017) m(6)A mRNA methylation controls T cell homeostasis by targeting the IL-7/STAT5/SOCS pathways. Nature 548, 338–342 10.1038/nature23450 28792938 PMC5729908

[39] Xie H., Li J., Ying Y., Yan H., Jin K., Ma X. et al. (2020) METTL3/YTHDF2 m(6) A axis promotes tumorigenesis by degrading SETD7 and KLF4 mRNAs in bladder cancer. J. Cell. Mol. Med. 24, 4092–4104 10.1111/jcmm.15063 32126149 PMC7171394

[40] Wen L., Pan X., Yu Y. and Yang B. (2020) Down-regulation of FTO promotes proliferation and migration, and protects bladder cancer cells from cisplatin-induced cytotoxicity. BMC Urol. 20, 39 10.1186/s12894-020-00612-7 32299393 PMC7164175

[41] Tao L., Mu X., Chen H., Jin D., Zhang R., Zhao Y. et al. (2021) FTO modifies the m6A level of MALAT and promotes bladder cancer progression. Clin. Transl. Med. 11, e310 10.1002/ctm2.310 33634966 PMC7851431

[42] Song W., Yang K., Luo J., Gao Z. and Gao Y. (2021) Dysregulation of USP18/FTO/PYCR1 signaling network promotes bladder cancer development and progression. Aging 13, 3909–3925 33461172 10.18632/aging.202359 PMC7906198

