# Supplementary material for: A new m6A methylation-related gene signature for prognostic value in patient with urothelial carcinoma of the bladder
Source: Biosci Rep. 2021 Apr 9;41(4):BSR20204456. doi: 10.1042/BSR20204456 (PMC8035626; doi:10.1042/BSR20204456)
Supplement: Supplementary Figure S1-S2 [file BSR-2020-4456_supp.pdf]

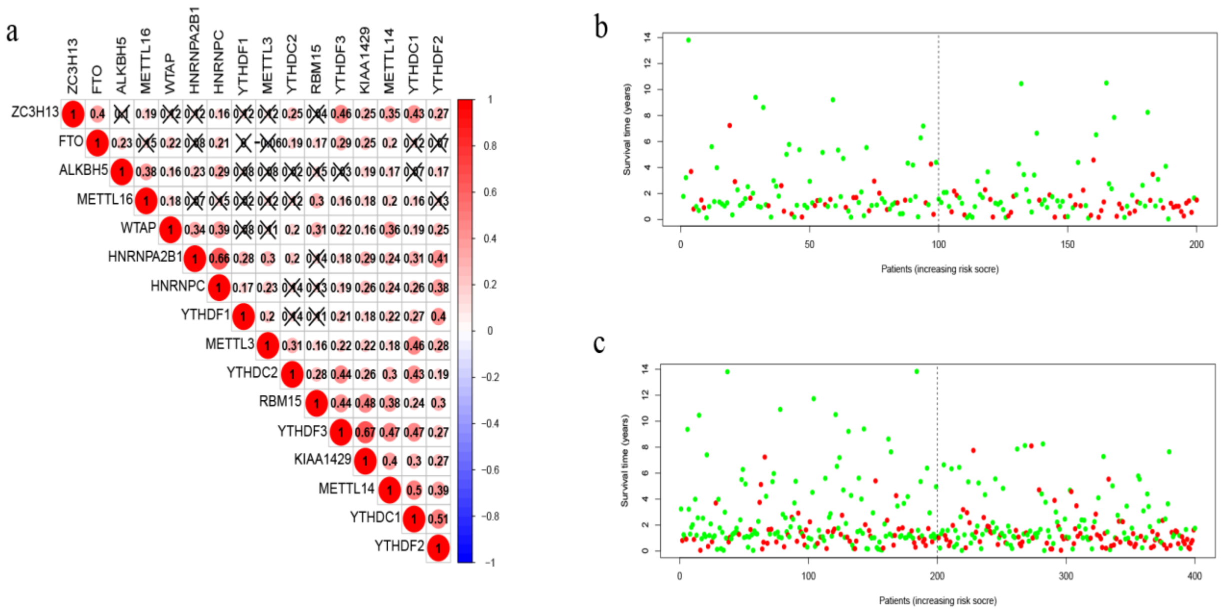

Fig. S1. The correlation analysis of the 16 m^{6}A RNA methylation genes**(a)** and distributions of risk scores **(b,c).**

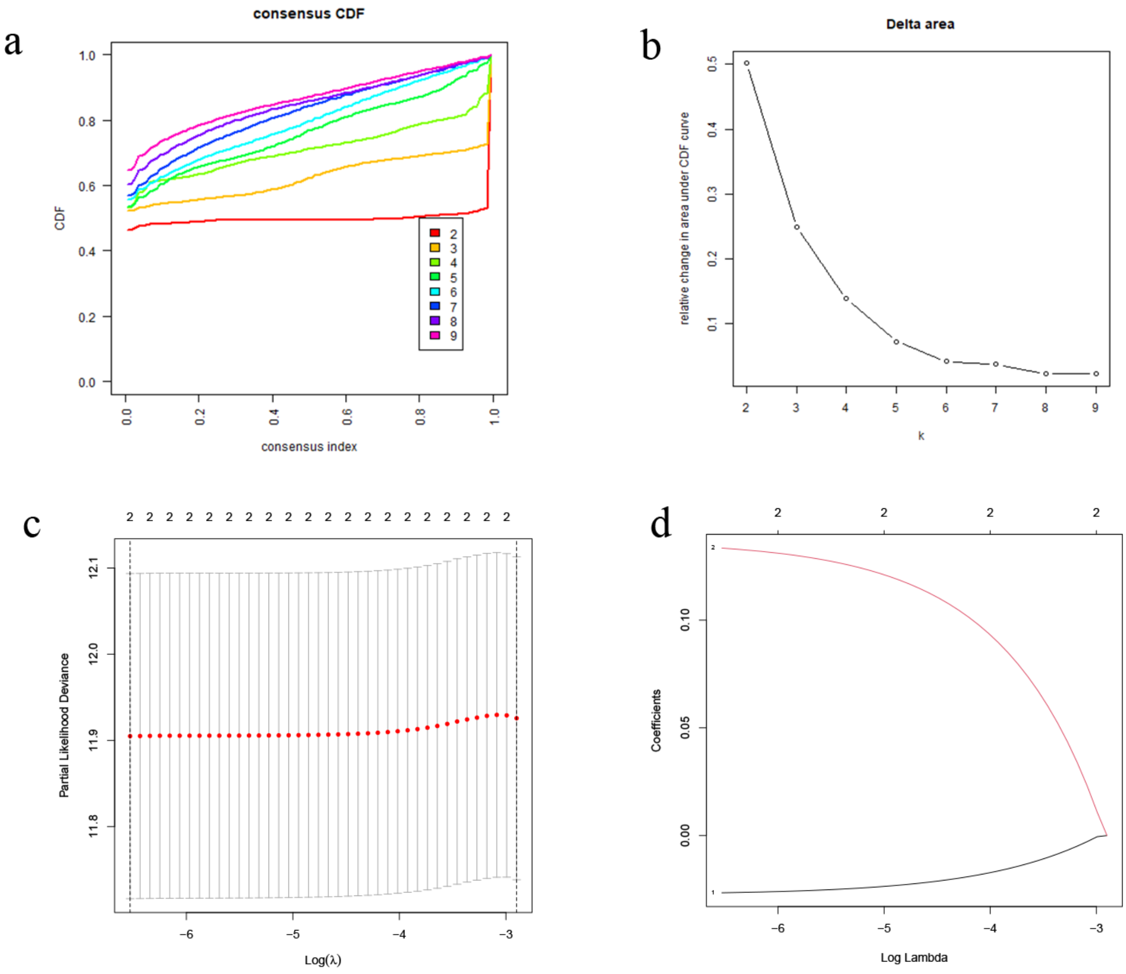

Fig. S2.Consensus clustering analysis and Lasso analysis in UC.a. The plot of cumulative distribution function (CDF) (k=2–9).**b.** Relative change in area under CDF curve (k=2–9);**c.** LASSO coefficient profiles of the expression of candidate genes. **d.** Selection of the penalty parameter in the LASSO model.
